# A New Article for Drying Cavities in Teeth, Preparatory to Filling

**Published:** 1852-01

**Authors:** 


*A New Article for Drying Cavities in Teeth, preparatory to Filling.*—In a communication recently received from Dr. Alphonso Severence, dentist, of Great Falls, N. H., the use of what is known by the name of *"flax cotton,"* is recommended as superior to any other substance for absorbing the moisture from cavities in teeth preparatory to filling. The Dr. sent us a small quantity with a request that we would give it a trial. We have done so, and find it to be a most admirable article for the purpose. It is easily applied to every part of the cavity on the point of an excavator, and absorbs the moisture more readily than unprepared raw cotton, and can be more conveniently employed than tissue paper—an article much used of late, by many dentists.

